# MVP (Micro Vascular Plug®) embolization of severe renal hemorrhages after nephrostomic tube placement

**DOI:** 10.1186/s42155-019-0087-8

**Published:** 2019-12-30

**Authors:** Francesco Giurazza, Fabio Corvino, Errico Cavaglià, Mattia Silvestre, Gianluca Cangiano, Francesco Amodio, Giuseppe De Magistris, Raffaella Niola

**Affiliations:** Vascular and Interventional Radiology Department, Cardarelli Hospital, Via Antonio Cardarelli 9, 80131 Naples, Italy

**Keywords:** Microvascular plug, Embolization, Renal hemorrages, Iatrogenic, Nephrostomy

## Abstract

**Background:**

We report our experience in managing iatrogenic renal bleedings after nephrostomic procedures by transarterial embolization using Micro Vascular Plug (MVP) (Medtronic, USA) as single or complementary embolization device with parenchimal sparing.

**Materials and methods:**

Five patients have been treated in a single center with transarterial embolization because of renal hemorrhages occurring after positioning of nephrostomic drainages.

All patients presented with back pain, severe hematuria and/or bright red blood into the nephrostomic bag, with fall in hemoglobin value.

After contrast enhanced CT scan confirming arterial active bleeding, rescue embolization was performed using MVP.

The renal parenchimal loss was estimated on final postembolization DSA. Creatinine values were monitored before and after the procedure.

**Results:**

Technical and clinical successes were obtained in all patients.

Two patients presented with extraluminal blush, one with multiple pseudoaneurysms, one with pseudoaneurysm with arterovenous fistula, one with extraluminal blush with arterovenous fistula.

MVP models were choosen oversized because of vasospasm that would underestimate the effective caliber of target vessel; MVP 3Q and MVP 7Q were adopted in one patient each, while MVP 5Q was released in three cases.

MVP was the sole embolizing agent in four patients; in one patient, MVP was employed after microcoils failed to obtain complete embolization.

The percentage of renal parenchimal lost was lower than 20%; no increase in Creatinine values was detected at dismission.

**Conclusions:**

According to proposed data, MVP seems to be a safe, effective and fast embolizing device that interventionalists could consider when facing renal bleedings, even as sole agent.

## Background

Percutaneous nephrostomy is a well-established procedure with a growing number of indications that can be grouped into three main categories: urinary drainage, urinary diversion and provision of access to the collecting system; the technical success rate of percutaneous nephrostomy catheter placement is high, ranging 84–99% (Pabon-Ramos et al. 2016), with the lowest rates in nondilated collecting systems.

The rate of minor and major complications grouped together is 10%; considering major hemorrhages only, those requiring blood transfusions occur in up to 4% of the cases while those requiring arterial embolization occur in up to 1% (Pabon-Ramos et al. 2016). Nephrectomy is reserved only for the cases in which the minimally invasive endovascular treatment fails (Summerton et al. 2012); actually the technical success of transarterial embolization of iatrogenic renal hemorrhages occurring during nephrostomic procedures is high (Jinga et al. 2013; Kervancioglu et al. 2014; Wang et al. 2015); multiple embolizing agents have been reported, mainly coils/microcoils, gelatin sponge and particles.

Here we report our experience in managing iatrogenic renal bleedings after nephrostomic procedures by transarterial embolization using Micro Vascular Plug (MVP) (Medtronic, USA) as single or complementary embolization device with parenchimal sparing.

## Materials and methods

### Sample data

All patients gave written informed consense to the procedure and data publication.

Five patients (3 men, 2 women; mean age: 49.6 years, range: 23–80 years) have been treated in a single center with transarterial embolization because of renal hemorrhages occurring after positioning of nephrostomic drainages (Table 1).

**Table 1:** Demographic details, target vessels caliber, angiographic pattern, MVP model, eventual other embolics adopted, pre- and post-embolization creatinine values

Patient	Sex	Age	Target Artery	Vessel caliber	Angiographic pattern	MVP model	Other embolics	Pre-embolization creatinine (mg/dL)	Post-embolization creatinine (mg/dL)
1	M	24	Interlobar branch of inferoanterior segmental artery	1.9 mm	Extraluminal blush	3Q	no	1.15	1.24
2	M	42	Interlobar branch of inferior segmental artery	3.2 mm	Pseudoaneurysms (3)	5Q	no	0.98	0.82
3	M	39	Inferior segmental artery	2.8 mm	Pseudoaneurysm with arterovenous fistula	5Q	Microcoils before plug	1.1	0.91
4	F	80	Inferior segmental artery	3.1 mm	Extraluminal blush	7Q	no	1.16	1.21
5	F	63	Interlobar branch of inferoposterior segmental artery	1.8 mm	Extraluminal blush and arterovenous fistula	5Q	no	4.31	3.27

*M* Male, *F* Female, *MVP* Micro vascular plug, *mg* Milligram, *dL* Deciliter

All subjects suffered from renal colic because of urinary stones, presenting with monolateral urinary tract obstruction and pielectasia; one patient had pyonephrosis also. Therefore 8.5–10 French pigtail nephrostomic drainages were positioned; this procedure has been performed by urologists under fluoroscopic guidance in an inpatient regimen. No patient was under anticoagulation/antiplatelet treatment at the moment of nephrostomic drainage; according to the guidelines for coagulation status and hemostasis risk in percutaneous interventions (Patel et al. 2012), International Normalized Ratio (INR) was < 1.5 and platelets count was > 50.000/μL.

Between 6 h and 72 h after the urologic procedure, patients presented with back pain and severe hematuria and/or bright red blood into the nephrostomic bag; all had a fall in hemoglobin value (more than 3 points); three of them required blood transfusion because of hemoglobin lower than 7 g/dl and presented with hemodynamic instability. The nephrostomy drainage was closed immediately after hemorrhage was suspected.

A contrast enhanced CT scan was acquired and signs of renal bleedings were evident: pseudoaneurysms, arterial extraluminal blush, venous fistula, perirenal or intracaliceal hematoma. Multiplanar reconstructions and maximum intensity projections were analyzed to properly plan the procedure and orientate the embolizing agent choice.

Patients were transfered to the angiosuite (Toshiba® Infinix I 8000C or Siemens® Artis Zee) and rescue embolizations performed by experienced interventional radiologists (> 5 years of experience in bleedings embolizations).

In four patients the procedures were performed through a right femoral access; in one case, because the patient suffered from neonatal tetraparesis, the only available peripheral access was the right humeral artery. After positioning under ultrasound guidance a 5Fr introducer, digital subtraction angiography (DSA) with power injector (flow: 5 ml/s, volume: 15 ml) of the involved renal artery was acquired to confirm CT findings; Cobra 1 or Simmons 1 catheter tips were adopted to access the origin of the renal artery. Three patients furthermore had a double renal artery. Definitive embolization was performed with the use of MVP, different size according to target vessel caliber measured on DSA images. Finally, DSA was repeated to confirm complete embolization.

The femoral accesses were closed with mechanical closure device (Angioseal 6VIP, Terumo®).

Renal parenchimal loss was estimated on final postembolization DSA compared to the renal parenchimography obtained at the beginning of the procedure.

Creatinine values were monitored before and after the embolization procedure in order to detect any renal functional damage.

### Micro Vascular Plug (MVP) features

The MVP is a mechanical embolization device; it presents a self-expanding nitinol skeleton ovoid-shaped covered with a Polytetrafluoroethylene (PTFE) coating (Giurazza et al. 2018).

Four sizes are commercially available: MVP3, 5, 7 and 9 that should be used to occlude vessels with a diameter up to 3 mm, 5 mm, 7 mm and 9 mm respectively.

MVP3 and MVP5 are delivered through microcatheter (0.027″), MVP7 is compatible with a 4Fr catheter while MVP9 requires a 5Fr catheter.

**Fig. 1:**
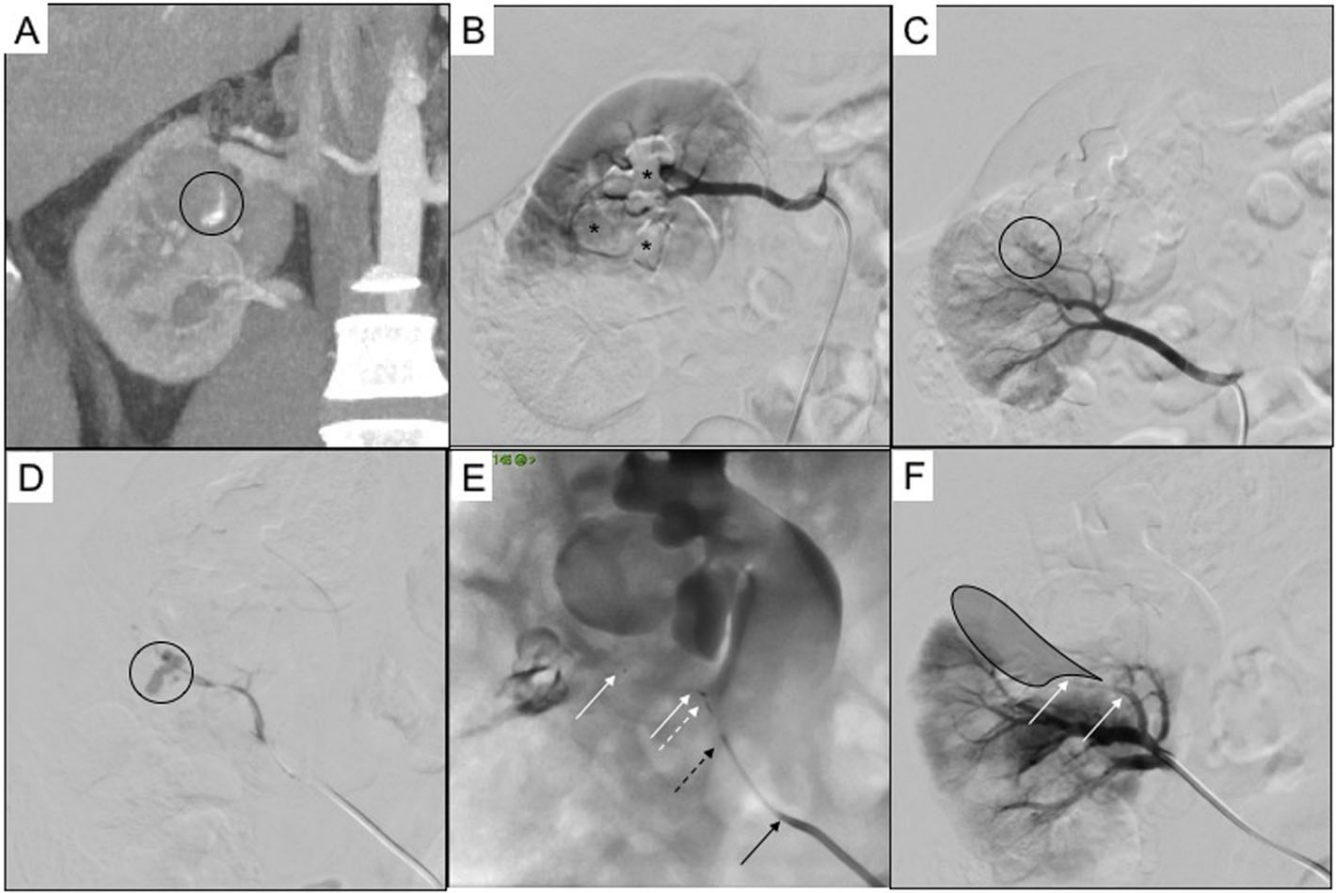
Patient 1. 24 years old man with double renal artery and urinary stones causing right pielectasia, treated with nephrostomy. **a** arterial phase CT scan reconstruction in coronal plane showing intracaliceal active bleeding (black circle); **b** DSA performed from the cranial renal artery showing no angiographic bleeding signs, previous CT contrast agent mixed with clots is appreciable in the calixs (black asterisks); **c** DSA performed from the caudal renal artery showing extraluminal blush (black asterisk) from the interlobar branch of the inferoanterior segmental artery; **d** superselective lobar microcatheterization confirming bleeding (black asterisk); **e** superselective embolization with MVP 3Q (white arrows indicate distal and proximal radiopaque markers of the MVP, white dotted arrow indicates the pusher wire radiopaque extremity after detachment, black dotted arrow indicates the 2.7Fr Progreat microcatheter, black arrow indicates the 5Fr diagnostic Cobra 1 catheter); **f** final DSA control showing no more angiographic bleeding (black shadow indicates the ischemic area, white arrows indicate distal and proximal radiopaque markers of the MVP)

**Fig. 2:**
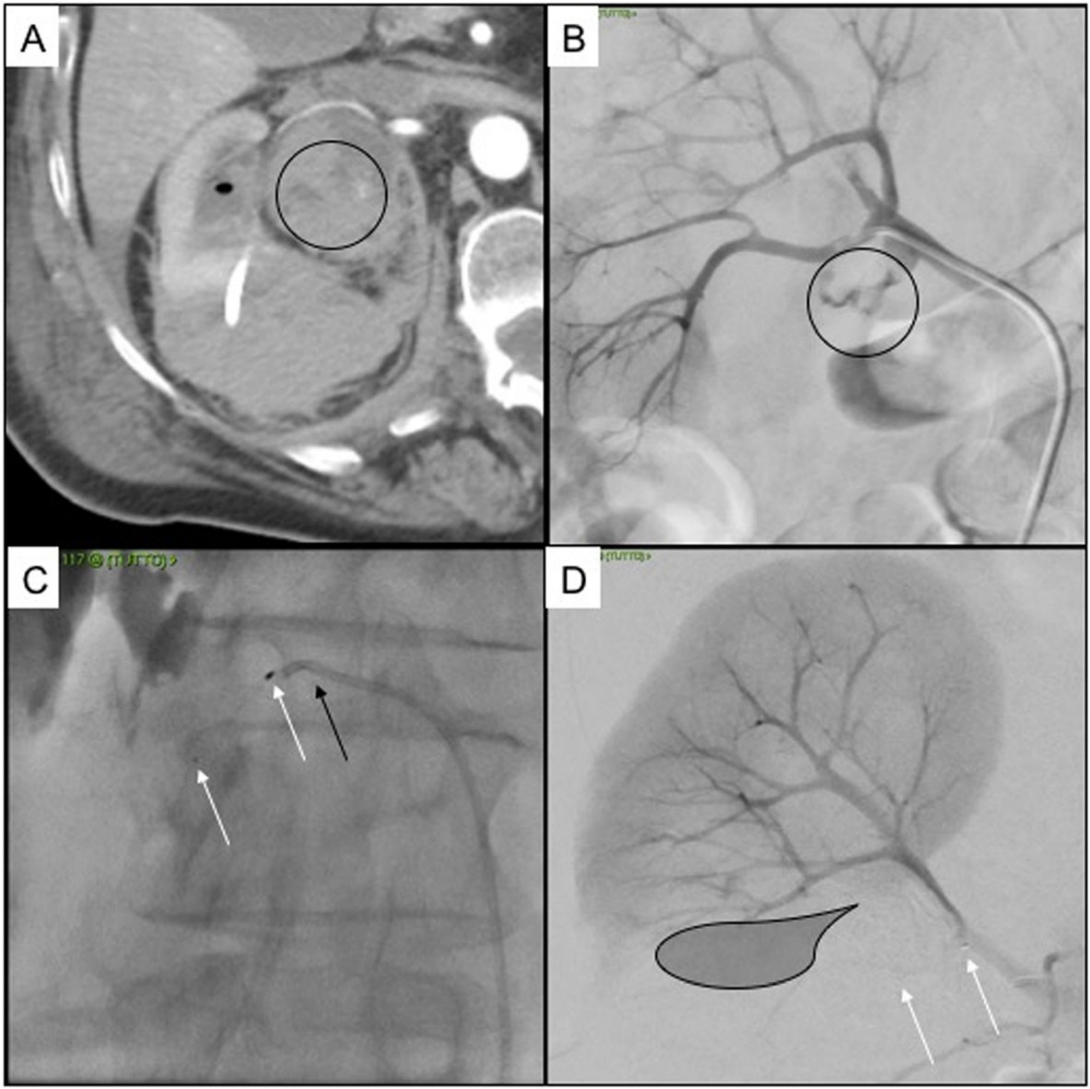
Patient 4. 80 years old female with urinary stones causing right pielectasia, treated with nephrostomy. **a** Axial CT scan in arterial phase showing active bleeding into the urinary pelvis (black circle), clots are appreciable into the calix (black ovoid), the nephrostomy drainage with perirenal hematoma is appreciable as well; **b** DSA showing extraluminal blush from the inferior segmental artery (black circle); **c** selective embolization with MVP 7Q (white arrows indicate distal and proximal radiopaque markers of the MVP, black arrow indicates the 5Fr diagnostic Cobra 1 catheter); **d** final DSA control showing no more angiographic bleeding (black shadow indicates the ischemic area, white arrows indicate distal and proximal radiopaque markers of the MVP)

**Fig. 3:**
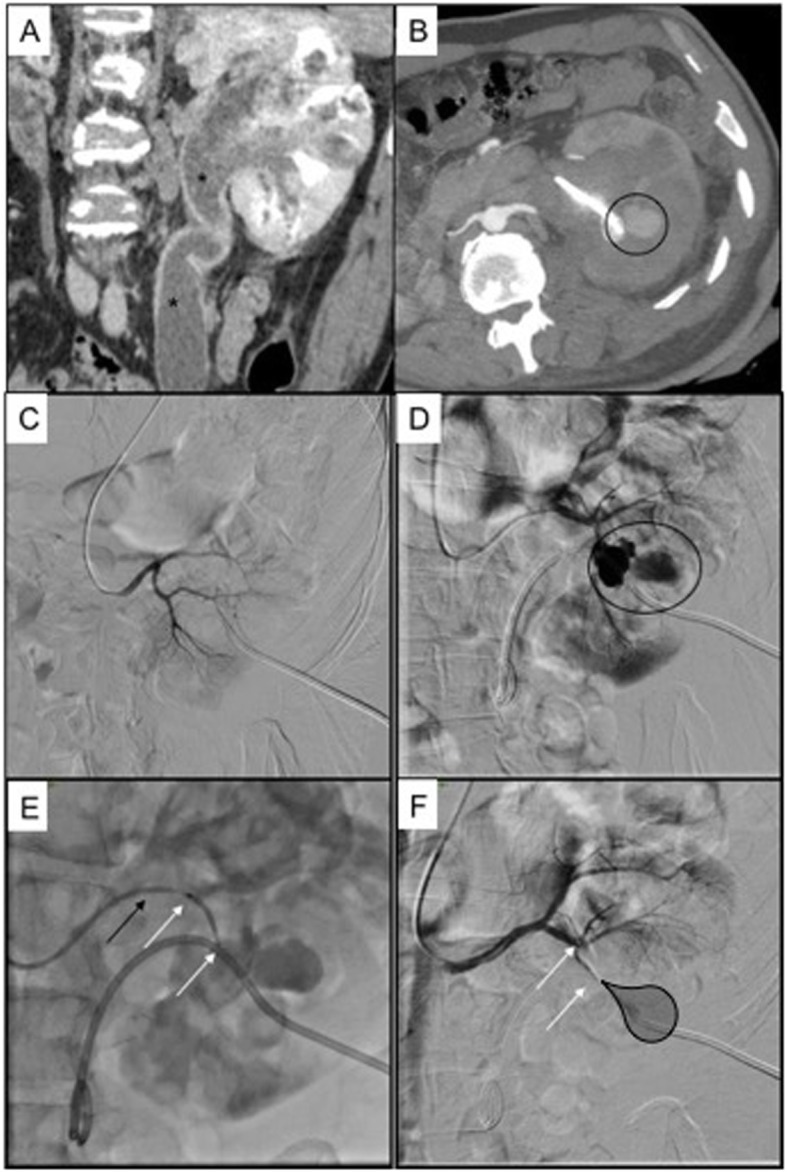
Patient 2. 42 years old man with double renal artery and urinary stones causing left pielectasia, treated with nephrostomy; this patient suffered from neonatal hypoxia with consequent tetraparesis, so the only vascular access available was right humeral artery. **a** prenephrostomy CT scan in coronal reconstruction showing severe ectasia of the pelvis and ureter (black asterisks); **b** postnephrostomy CT scan in arterial phase showing a pseudoaneurysm adjacent to the nephrostomic drainage (black circle); **c** DSA performed from the cranial renal artery showing no angiographic bleeding signs; **d** DSA performed from the caudal renal artery showing three pseudoaneurysms (black circle) from the interlobar branch of the inferior segmental artery; **e** selective embolization with MVP 5Q (white arrows indicate distal and proximal radiopaque markers of the MVP, black arrow indicates the 5Fr diagnostic Cobra 1 catheter); **f** final DSA control showing no more angiographic bleeding (black shadow indicates the ischemic area, white arrows indicate distal and proximal radiopaque markers of the MVP)

The MVP is soldered to a pusher wire and the detachment is mechanical with anticlockwise torque. Each MVP presents two radiopaque markers at proximal and distal extremities that allows proper visualization in fluoroscopy (Figs. 1, 2 and 3) and at CT scan.

Once delivered, MVP3 and MVP5 present a length of 12 mm while MVP7 and MVP9 present a lenght of 16 mm. This is a relevant aspect to consider when choosing the MVP model because the embolization property depends by the presence of a straight landing zone able to receive the whole length of the plug; empirically, if the MVP is released in a tortuous landing zone, the embolizing effect is not obtained because the nitinol skeleton does not expand properly. Another technical shrewdness is flush the whole catheter/microcatheter dead space with saline just before introducing the MVP through the hub, in order to avoid clot formation that may hinder the release.

## Results

Technical success, intended as disappearance of pathological angiographic findings, was demonstrated in 5/5 patients at last DSA; accordingly, clinical success, intended as restoration of hemoglobin values and hemodynamic stability, was achieved in all subjects.

The iatrogenic damage involved the interlobar branch of the inferoanterior segmental artery (1 patient), interlobar branch of the inferior segmental artery (1 patient), the inferior segmental artery (2 patients) and the interlobar branch of the inferoposterior segmental artery (1 patient); these vessels measured at DSA between 1.9 mm and 3.2 mm.

At pre-embolization DSA, two patients presented with an extraluminal blush (Figs. 1 and 2), one with multiple pseudoaneurysms (Fig. 2), one with a pseudoaneurysm with arterovenous fistula, one with an extraluminal blush with arterovenous fistula.

MVP model was choosen oversized in all patients because of the vasospasm that would underestimate the actual vessel caliber (Table 1); the oversize rate was selected empirically up to 50% of the vessel caliber, according to the degree of the vasospasm appreciable at DSA. MVP was the sole embolizing agent in four patients; in Patient 3, MVP was released after microcoils failure to obtain complete embolization because of inappropriate sizing.

MVP 3Q and 5Q were delivered through a 2.7 Fr microcatheter (Progreat, Terumo®); MVP 7Q was released through a 5Fr catheter (Merit Medical®). All MVPs detached at first attempt without technical obstacles and no device migration occurred.

The nephrostomy was not routinely removed before the procedure.

The percentage of renal parenchimal ischemic area was lower than 20% in all cases.

No increase in creatinine values were detected at dismission compared to pre-embolization data (Table 1). In Patients 1, 2, 3 and 4 the post-embolization values remained in a normal range, considering 1.3 mg/dL the upper limit in adult population, and did not show relevant variations. Patient 5 was a dialytic subject and so the post-procedural creatinine value, even if did not increase, was influenced by the post-embolization dialysis.

According to CIRSE classification (Filippiadis et al. 2017), no complications occurred concerning the embolization procedures.

## Discussion

The first use of MVP in the clinical practice has been reported in literature in 2014 (Pellerin et al. 2014); since then, series concerning MVP embolization in multiple anatomical districts described successful results (Kleine et al. 2015; Carlson et al. 2017; Burkhardt et al. 2018; Giurazza et al. 2018; Pellerin et al. 2014; Shwe et al. 2014; See et al. 2017; Boatta et al. 2017; Conrad et al. 2015; Ratnani et al. 2019; Mahdjoub et al. 2018; Duvnjak et al. 2018; Bailey et al. 2019, Barrett et al. 2018; Boudjemline 2017; Wang-Giuffre and Breinholt 2017; Sathanandam et al. 2017a, b; Hao et al. 2016; Chick et al. 2017; Bhardwaj et al. 2017). In neuroendovascular embolization procedures, its use has been successfully reported in intracranial aneurysms and acute hemorrhages (Kleine et al. 2015; Carlson et al. 2017; Burkhardt et al. 2018; Giurazza et al. 2018), high-flow carotid cavernous (Shwe et al. 2014) and basilar arterio-venous fistula (See et al. 2017), vein of Galen malformation (See et al. 2017). On the other hand, in extraneurovascular embolization, its application has been reported with good clinical and technical outcomes in pulmonary arteriovenous malformations (Boatta et al. 2017; Conrad et al. 2015; Ratnani et al. 2019; Mahdjoub et al. 2018; Duvnjak et al. 2018; Bailey et al. 2019; Barrett et al. 2018), in hepatic, genicular, epigastric and genicular arteries bleeding (Giurazza et al. 2018) as well as in bleeding ectopic duodenal varix along with transjugular intrahepatic portosystemic shunt (Bhardwaj et al. 2017). Some authors presented also safe and effective experiences in paediatric patients where the MVP has been choosen to treat congenital heart disease, as closure of patent ductus arteriosus closure, Blalock-Taussig shunt, veno-venous and coronary fistulas, (Boudjemline 2017; Wang-Giuffre and Breinholt 2017; Sathanandam et al. 2017a, 2017b) and life-threatening upper gastrointestinal bleeding (Hao et al. 2016). Table 2 summarizes the published cases in literature concerning the MVP usage for the management of hemorrhage. Finally, a case report has described the use of the MVP in lymphatic embolization of thoracic duct leak (Chick et al. 2017).

**Table 2:** Published cases in literature concerning the MVP usage for the management of hemorrhage

Authors	Year of publication	N° of Patients	Vessels embolized	MVP embolization success
Giurazza et al.	2018	6	External carotid, hepatic, renal, epigastric (2), genicular aa.	100%
Bhardwaj et al.	2017	1	Duodenal varix	100%
Hao et al.	2016	1	Gastroduodenal a.	100%
Kleine et al.	2015	4	Facial, thyroid, vertebral (2) aa.	75%

*N°* Number, *aa* Arteries, *MVP* Micro vascular plug

Concerning renal bleeding embolization with MVP, only one case has been described in literature (Giurazza et al. 2018); the presented series focuses specifically on iatrogenic renal hemorrhages embolization; being the renal parenchima widely arterialized, the angiographic spectrum of vascular lesions includes vessel laceration with extraluminal blush, arterovenous or arterocaliceal fistulas and one or more pseudoaneurysms. The authors found the MVP particularly indicated in this anatomical district because the division branches of the renal artery present a straight course and so can properly receive the MVP; the preprocedural CT findings, confirmed by the diagnostic DSA, play a crucial role in the choice of the procedural strategy and orientate the embolizing agents adopted. Furthermore thanks to the possibility of retrieving and repositioning the MVP, the embolization can be performed precisely and selectively in order to spare the largest possible portion of the healthy parenchima and preserve renal functional status, as demonstrated by the creatinine values at dismission. Compared to detachable coils, in this scenario MVP presents some advantages; first, the procedural time is shorter because the device release is faster and, if properly selected, one MVP is enough to obtain immediate interruption of blood flow because of its architecture; then, the risk of occluding non target area is minimized because the MVP positioning is highly predictable; finally, thanks to the PTFE coating, the MVP has an effective embolizing action also in patients with coagulation alterations. Because of the terminal renal vascularization, particulated and liquid embolics are not preferred to avoid the risk of non target ischemic embolizations.

In this sample, in three patients the injured kidney was vascularized by more than one renal artery originating from the aorta; this condition represents a natural protective factors in case of renal hemorrhage and it is appreciable in approximately the 20% (Majos et al. 2018) of the general population.

The authors have choosen oversized MVP up to 50% compared to the target vessel diameter; this because all the procedures have been performed in patients with active bleeding and so vasoconstriction response may underestimate the proper vessel caliber on angiographic images.

The MVP should not be considered as an alternative to coils; it is instead a device with different features that could be used apart from or together with coils as complementary agent for backward sealing (Giurazza et al. 2018), as occurred in one patient in this series.

In renal bleedings embolization, the MVP presents the relevant advantage of distal navigation thanks to the release through microcatheters and 4-5Fr catheters that allows selective and parenchimal sparing embolization: furthermore the detachable system and the soft nitinol skeleton permit to retract and repositioning MVP multiple times until the operator is satisfied. The main limitation of MVP application in renal embolization is the availability of a sufficient straight landing zone able to receive the unconstrained length of the MVP.

Because of the diversified arteriographic presentation of acute renal hemorrhage, proper selection of the embolic agent is a key to obtain successful hemostasis.

## Conclusions

Even if the reported experience is limited because of the small sample size, according to the proposed data MVP seems to be a safe, effective and fast embolizing device that interventionalists could consider when facing renal bleedings, even as sole embolizing agent.

## Data Availability

The dataset supporting the conclusions of this article is available at Cardarelli Hospital RIS-PACS system; for any questions, please contact the corresponding author.

## Abbreviations

CIRSE: Cardiovascular and Interventional Radiology Society of Europe
CT: Computed Tomography
DSA: Digital subtraction angiography
Fr: French
INR: International Normalized Ratio
MVP: Microvascular plug
PTFE: Polytetrafluoroethylene
